# Role of Redox Signaling and Inflammation in Skeletal Muscle Adaptations to Training

**DOI:** 10.3390/antiox5040048

**Published:** 2016-12-13

**Authors:** Maria Carmen Gomez-Cabrera, Jose Viña, Li Li Ji

**Affiliations:** 1 Department of Physiology, Fundacion Investigacion Hospital Clinico Universitario/INCLIVA, University of Valencia, València 46010, Spain; jose.vina@uv.es; 2 Laboratory of Physiological Hygiene and Exercise Science, School of Kinesiology, University of Minnesota, 1900 University Avenue, Minneapolis, MN 55455, USA; llji@umn.edu

**Keywords:** oxidative stress, anti-inflammatories, hypertrophy, protein synthesis, prostaglandins

## Abstract

The inflammatory response to exercise-induced muscle damage has been extensively described. Exercise has important modulatory effects on immune function. These effects are mediated by diverse factors including pro-inflammatory cytokines, classical stress hormones, and hemodynamic effects leading to cell redistribution. As has been reported regarding oxidative stress, inflammation can have both detrimental and beneficial effects in skeletal muscle. In this review we will address the role of inflammation on protein metabolism in skeletal muscle. Specifically, we will review studies showing that treatment with cyclooxygenase-inhibiting drugs modulate the protein synthesis response to one bout of resistance exercise and to training. Understanding how these drugs work is important for the millions of individuals worldwide that consume them regularly. We will also discuss the importance of reactive oxygen species and inflammatory cytokines in muscle adaptations to exercise and the Janus faced of the use of antioxidant and anti-inflammatory drugs by athletes for optimizing their performance, especially during the periods in which muscle hypertrophy is expected.

## 1. Introduction

Inflammation and its impact have received increasing attention by the scientific community in the last decade, being associated with fundamental physiological processes (e.g., cell signaling) and being implicated in almost all ageing-related degenerative diseases. To emphasize the importance of the topic, the Journal “Science” dedicated a special issue to inflammation in 2013, with the opinions suggesting that the study of inflammation must be at the center of research interest both at the scientific and the social level in the future [1].

Redness, heat, swelling, and pain are the hallmarks of inflammation described by Celsus 2000 years ago [1]. Inflammation is a physiological response of the immune system to harmful stimuli, such as irritants, pathogens, and damaged cells. It is mediated by a variety of soluble factors, including a group of secreted polypeptides known as cytokines. Cytokines are important molecules in cell signaling and are regulators of host immune responses to infection, inflammation, and trauma [2]. They are divided in two main categories, namely pro-inflammatory and anti-inflammatory cytokines [3]. Pro-inflammatory cytokines (e.g., tumor necrosis factor (TNF)-α, interleukin (IL)-1, and IL-6) are generally produced at the site of infection or trauma where tissue damage or destruction occurs. Pro-inflammatory cytokines contribute to the regeneration of a healthy tissue as they trigger the degeneration and clearance of damaged or infected cells. The anti-inflammatory cytokines (e.g., IL-8 and IL-10) are a series of regulatory molecules that control the pro-inflammatory cytokine response. Under physiological conditions, anti-inflammatory cytokines serve as immunomodulatory elements that limit the potentially injurious effects of sustained or excess inflammatory reactions and promote tissue regeneration [2]. However, under pathological conditions, they may either provide insufficient control over pro-inflammatory activities or overcompensate the immune response, rendering the host at risk from pathogens [4]. Thus, there must be a tight equilibrium between pro-inflammatory and anti-inflammatory response to optimize inflammation as a defensive mechanism of the immune system. The symptoms of acute inflammation dissipate rapidly, and though inconvenient, are also reassuring, as they represent the immune system in action. The immune system clears the infection, guides the repair of damaged tissue and the symptoms disappear. However, although inflammation is so beneficial during an infection, it can also contribute to the pathogenesis of several age-related chronic diseases, such as neurodegenerative disease, cardiovascular disease, and metabolic disease. In fact, in older people, a chronically-elevated endocrine, pro-inflammatory environment, termed inflammaging, has several negative implications for health and wellbeing. It is characterized by elevated levels of serum pro-inflammatory cytokines and reduced levels of anti-inflammatory cytokines [5]. In this review we aim at highlighting the beneficial, but also in some cases detrimental, role of inflammation in skeletal muscle. The inflammatory response to exercise-induced muscle damage has been extensively described [6]. Exercise has important modulatory effects on immunocyte dynamics, and possibly on immune function. These effects are mediated by diverse factors, including exercise-induced release of pro-inflammatory cytokines, classical stress hormones, and hemodynamic effects leading to cell redistribution [7]. There is evidence in the literature on the implication of several cytokines in the pathogenesis of muscle wasting and sarcopenia, most notably TNF-α, but also IL-1β, IL-6, interferon (IFN)-γ, and transforming growth factor (TFG)-β [8]. However, the beneficial role of inflammation in protein metabolism in skeletal muscle during exercise has also been highlighted [9]. We will review paradoxical studies showing that treatment with inhibitors of prostaglandin (PG) synthesis blunts the protein synthesis response to an acute bout of resistance exercise [9,10], but does not have interfering effects on the muscle adaptations to resistance exercise training [11]. In fact, in older individuals, a substantial enhancement of muscle mass and strength has been observed when cyclooxygenase (COX) inhibitors are used in combination with resistance exercise training [12]. Finally, we will try to establish the connection between oxidative stress and inflammation in exercise. We will discuss the importance of reactive oxygen species (ROS) and inflammatory cytokines in muscle adaptations to exercise training and the Janus faced of the use of antioxidant and anti-inflammatory drugs by the sport population.

## 2. Exercise-Induced Inflammation and Oxidative Stress in Skeletal Muscle

**Figure 1:**
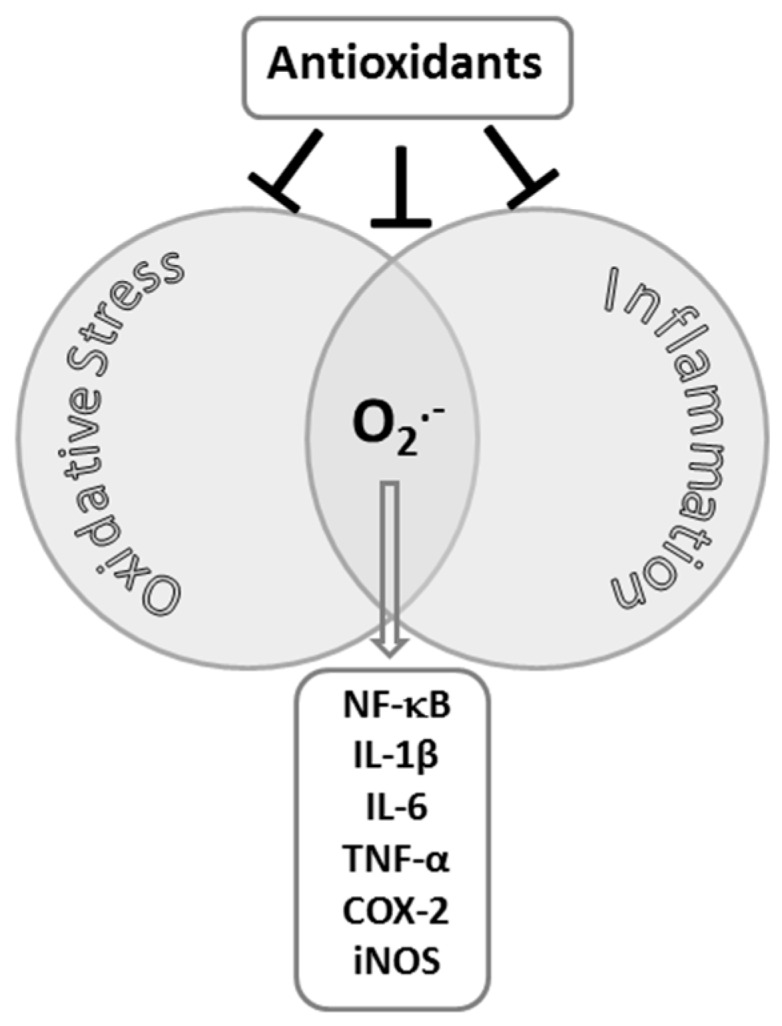
Interactions between oxidative stress and inflammation.

Unaccustomed exercise may result in significant damage to skeletal muscle and cause delayed onset muscle soreness. Two physiological responses, inflammation and oxidative stress, have been proposed to explain how exercise initiates damage to skeletal muscle fibers [13]. There are several parallels between the effects of chronic inflammation and oxidative stress in skeletal muscle. First, evidence showing that free radicals and inflammation are related was published in 1980 [14]. The participation of superoxide (O_2_^•−^) in the inflammatory response was inferred by the anti-inflammatory effect of the parenterally-administered antioxidant enzyme superoxide dismutase (SOD) [14,15]. The toxicity of O_2_^•−^, or the oxidant species derived from it, was assumed to account for the anti-inflammatory activity of SOD [16] (See Figure 1). However, it was also suggested that O_2_^•−^, per se, is relatively innocuous and that the role of O_2_^•−^ in inflammation could not be due solely to direct cytotoxicity but to the generation of a chemotactic factor for neutrophils [14]. Subsequent evidences indicate that the activation of redox-sensitive transcription factors, such as nuclear factor κB (NF-κB), may modulate the gene expression of key players involved in the inflammatory process, IL-1β, IL-6, TNF-α, (COX)-2, adhesion molecules, and inducible nitric oxide (NO) synthase (iNOS) (See Figure 1) [17]. NF-κB is a dimeric transcription factor composed of members of the Rel family [18]. When activated, NF-κB migrates to the nucleus and binds to specific sites in the promoter sequences of many genes. NF-κB is activated by a variety of external stimulants, such as ROS and cytokines.

Several genes require NF-κB binding to activate its transcription. Among them, we can highlight manganese superoxide dismutase (MnSOD), iNOS, COX-2, γ-glutamylcysteine synthetase (GCS), vascular cell adhesion molecule-1 (VCAM-1), and cytokines. These genes are involved in a wide variety of biological functions such as antioxidant, inflammation, immunity, and apoptosis [19].

Hollander et al. [20] first reported that one bout of exhaustive exercise can activate MnSOD gene expression in rat skeletal muscle, due to enhanced NF-κB binding in muscle nuclear extracts. We found that NF-κB could be activated in a redox-sensitive manner during muscular contraction and that the highest levels of NF-κB binding were observed at 2 h post exercise in rat skeletal muscle [21]. From this study we concluded that ROS activate a cascade of intracellular events that lead to an elevated gene expression of MnSOD, as reported earlier [22]. It was hypothesized that ROS-induced oxidation of cysteine residues in the upstream activators of NF-κB could trigger inflammation [23].

Muscle fiber damage due to strenuous exercise, especially lengthening contraction, can trigger the release of inflammatory cytokines from immune cells and/or damaged muscle tissues. Blood-borne polymorphonuclear (PMN) plays a critical role in defending the cell from viral and bacterial invasion by activating reduced nicotinamide adenine phosphate (NAD(P)H) oxidase to produce ROS via a respiratory burst [24]. During the early phase of muscle injury, inflammatory cytokines promote the gene expression of adhesion molecules such as VCAM-1, cytokine-induced neutrophil chemoattractant-1 (CINC-1), monocyte chemoattractant protein-1 (MCP-1), and NO. In addition, some cytokines can bind with membrane receptors and activate specific ROS-generating enzymes, such as NAD(P)H oxidase, and xanthine oxidase (XO) [6]. Endothelial cells from injured muscle are known to secrete TNF-α, IL-1, IL-6, and IL-8, providing a positive feed-forward cycle [25]. Thus, oxidative stress and inflammation are closely linked.

It has been shown that the use of antioxidants can modulate inflammation in skeletal muscle (see Figure 1). Aoi et al. [26] first showed that hydrogen peroxide (H_2_O_2_) stimulated NF-κB activation and expression of CINC-1 and MCP-1 in myotubes and that it was prevented with α-tocopherol. Similar results were found in an in vivo study in which a high vitamin E diet prevented the exercise-induce increase in CINC-1 and MCP-1 levels and nuclear p65 content in rat gastrocnemius muscle [27]. More recently the use of vitamin C [28], flavonoids and anthocyanins [29,30] have been tested as potential treatments to reduce the o**x**idative stress and inflammatory responses after exhaustive exercise to accelerate recovery [31]. Glutathione homeostasis is also known to be an important factor in muscle inflammation and the main factor regulating intracellular reduced glutathione (GSH) level is GCS activity. High levels of GSH prevent the inflammatory process partially by inhibiting intercellular adhesion molecule 1 (ICAM-1) expression [32]. Interestingly GSH content has been reported to be higher in rabbit tibialis muscle 24 h after an isokinetic stretch injury, accompanied with elevated glutathione peroxidase (GPX) and glutathione reductase (GR) activities [33]. Since an optimal reduced to oxidized glutathione (GSH:GSSG) ratio is crucial in redox signaling, these studies confirm that there is a clear parallel between oxidation and inflammation in skeletal muscle cells.

To determine muscle damage, serum levels of skeletal muscle enzymes are quantified. Creatine kinase (CK), lactate dehydrogenase, aldolase, myoglobin, troponin, and aspartate aminotransferase are the most useful serum markers of muscle injury. We have shown in weight-lifting [34], cycling [35], marathon running [36], and soccer [13], that the inhibition of XO, a free radical-generating enzyme activated during muscle contraction, can significantly decrease the exercise-induced muscle damage and oxidative stress in a variety of sports. However, other research groups report variable findings when using antioxidant supplements to prevent exercise-induced muscle damage [37]. In the same way, the effects of anti-inflammatory drugs on markers of muscle damage are also controversial [37]. Possible reasons for these variations include differences in the dose and biological activity of individual antioxidants and anti-inflammatory drugs [38]. Anti-inflammatory drugs exert their effect on inflammatory reactions through a number of pathways, including the inhibition of PG synthesis, neutrophil adhesion and activation [38]. Bourgeois and co-workers found that naproxen sodium administration did not alter CK rise or muscle force deficit, nor perceived muscle pain in healthy subjects after a resistance exercise protocol [39]. On the contrary, a prophylactic dose of ibuprofen decreased muscle soreness perception and assisted in restoring muscle function after a strenuous eccentric exercise bout. However, no prevention in CK release from muscle was reported in this study [40]. Pizza and co-workers found that ibuprofen administration reduced CK activity but not the neutrophil response or other indirect markers of muscle injury during recovery from eccentric arm exercise [41]. Finally, administration of diclofenac significantly reduced quantitative indices of exercise-induced skeletal muscle damage (CK) in humans [42], and ketoprofen treatment after muscle-damaging exercise reduced muscle soreness and improved force recovery [43]. The variable research findings reflect differences in sampling time, the extent of muscle damage after exercise, and training status of subjects, in addition to the dose, timing, and type of drug administration [38].

We have recently reviewed the role of ROS in muscle adaptations to exercise training [44,45]. As previously mentioned, treatment with different types of antioxidants may cause beneficial effects when administered before one single session of exhaustive exercise. They have been involved in the prevention of muscle damage [13,35,36], oxidative stress [46,47,48,49], muscle fatigue [50,51,52], and the decrease in muscle performance associated to ROS production during one bout of exhaustive exercise [53,54,55]. Based on this data, a significant number of athletes, especially elite athletes, consume dietary antioxidant supplements seeking beneficial effects on performance. However, during the past years several studies have clearly indicated that ROS activate important cell signaling pathways that mediate skeletal muscle adaptations to exercise such as mitochondrial biogenesis [56,57,58], the induction of the endogenous antioxidant defense [59], and hypertrophy [60], among others [43]. The fact that ROS play a role in promoting adaptations in muscles represents a significant change in biological understanding whereby, in the past, it was assumed that even low doses of ROS were universally harmful. Thus, by ingesting high doses of antioxidant vitamins on a regular basis in an attempt to enhance muscle performance, individuals may actually be retarding, or even hampering, the adaptations to exercise training [61].

The question of whether anti-inflammatory drugs can also act as a double-edged sword in skeletal muscle during exercise will be discussed in the next section.

## 3. Role of Inflammation in Skeletal Muscle Adaptations to Exercise: Should We Block It with Anti-Inflammatory Drugs?

Improvement of muscle mass is of great interest to many populations, including the sport population [62]. Athletes and body builders desire to increase muscle mass and strength for competitive reasons. Older adults are also interested in maintaining their muscle mass and function. Sarcopenia, defined as the involuntary loss of skeletal muscle mass and strength is a debilitating clinical condition associated with old age [63].

Whether a muscle atrophies or hypertrophies depends on the balance between the rates of protein synthesis and degradation [10]. A debate exists on whether muscle disuse is explained by a decrease in protein synthesis or, on the contrary, an increase in protein degradation [64]. A review of the literature shows that both aspects of muscle protein homeostasis are disrupted by prolonged disuse. Proteolysis increases and synthesis declines. These changes have additive effects, but it seems that proteolysis plays the most important role in muscle atrophy [64].

**Figure 2:**
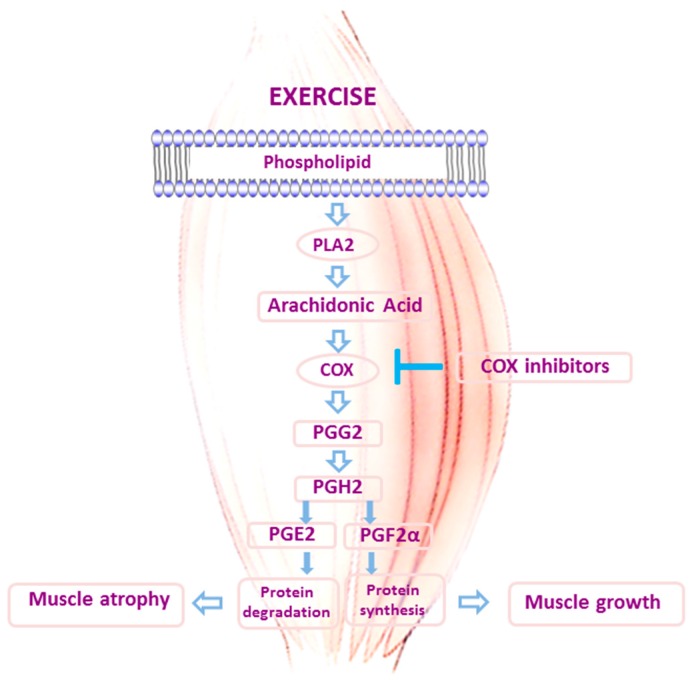
Schematic representation of the regulation of skeletal muscle protein metabolism through the COX pathway.

The role of inflammation in the regulation of the muscle mass has been an object of study [11]. It is well established that PGs participate in the inflammatory responses. PGs were discovered in 1936 when it was found that seminal fluid and seminal vesicles from most animals, including humans, contain a substance which causes contraction of the smooth muscle of the uterus [65]. PGs are formed from unsaturated fatty acids, primarily arachidonic acid. Arachidonic acid is degraded and liberated from the membrane of cells by phospholipase A_2_ (PLA_2_). The enzyme prostaglandin-endoperoxide synthase, more commonly known as COX, is a dual-functioned enzyme that converts arachidonic acid to prostaglandin G_2_ (PGG_2_) and then prostaglandin H_2_ (PGH_2_) [66], which is then rapidly converted to specific PGs (e.g., PGD_2_, PGE_2_, PGF_2α_, and PGI_2_) by PG synthases [11]. The COX inhibitors, ibuprofen, aspirin, and acetaminophen represent the most commonly consumed classes of drugs in the world [67] (See Figure 2).

The PGs and their related substances, which include thromboxane, prostacyclin, and leukotrienes, function in the defence of cells against sudden changes (trauma, disease, or stress) thereby maintaining or reconstituting the normal function [11]. The PGs are involved in a large number of physiological processes such as the homeostatic maintenance of the gastrointestinal tract (PGs reduce secretion of gastric acid and modulate gastric mucosal blood flow), renal function (PGs are natriuretic, influence renal blood flow, glomerular filtration rate, and the release of renin), blood clotting, embryonic implantation, and parturition [68]. Thus, if the formation of PGs is blocked, for instance, a peptic ulcer can be rapidly formed and the renal function deteriorates due to an uncontrolled action of angiotensin [68]. However, PGs participate also in the induction of fever, in the inflammatory responses and in pain [10].

COX-1 and COX-2 activity increase in human skeletal muscle with resistance exercise [69]. The first studies of PGs production and release by skeletal muscle in response to muscular work were published in 1974 [70]. Subsequent studies found that the muscles could produce PGD_2_, PGE_2_ (the predominant PG produced), PGF_2α_, and PGI_2_ [71]. In 1982 it was found that PGE_2_ and PGF_2α_ could be involved in the regulation of protein balance in skeletal and cardiac muscles [10]. PGE_2_ increases protein degradation through the induction of intramuscular IL-6 and the proteolytic muscle ubiquitin ligase RING finger protein-1 (MuRF-1) [11], whereas PGF_2α_ stimulates protein synthesis through the activation of mammalian target of rapamycin (mTOR) signalling in skeletal muscle via a PI3K/ERK-dependent pathway [72]. Rodemann and Goldberg found that arachidonic acid supplementation exerted muscle production of both PGs, but about twice as much PGE_2_ was synthesized compared with PGF_2α_ [10]. Thus, the net effect of arachidonic acid was a catabolic one in different muscles types (rates of protein degradation increased between 20% and 40%). However, in the soleus muscle the enhanced proteolysis was accompanied by a 50% increase in protein synthesis, although protein degradation increased in absolute terms more than protein synthesis [10]. In an in vitro study with C2C12 skeletal myocytes, arachidonic acid supplementation stimulated PG release and skeletal muscle cell hypertrophy via a COX-2-dependent pathway [73]. Interestingly the effects of arachidonic acid are blocked by different inhibitors of COX [10].

PGs production in skeletal muscle is dependent on exercise intensity and duration [11]. Low-level muscular work does not increase PGE_2_ levels [74] whereas resistance exercise stimulates the muscle to produce PGE_2_ and/or PGF_2α_ during and/or after exercise [74]. Furthermore, increasing the workload during aerobic exercise increases the muscle production of PGE_2_ [75]. COX inhibitors can significantly reduce the resting levels and the exercise-induced increments in skeletal muscle PGE_2_ [76,77]. Moreover, it has been found that ibuprofen treatment blunts early translational signalling (MEK-ERK) responses in human skeletal muscle following resistance exercise suggesting that PGs are important signalling molecules during early post-exercise recovery [78]. In 2002 Trappe and co-workers found that treatment with ibuprofen (1200 mg/day) and acetaminophen (4000 mg/day) attenuated the increased rate of muscle protein synthesis 24 h after high-intensity eccentric resistance exercise [9]. These results questioned the extended use of anti-inflammatory drugs among the sport population to relieve the muscle pain after exercise. Since then, several animal and human studies have been developed to determine the chronic effects of COX inhibitors on exercise induced adaptations. A summary of the main findings reported in the human studies is shown in Table 1.

Surprisingly, chronic consumption of COX inhibitors during exercise training does not appear to interfere with the muscle mass and strength gains expected from typical resistance exercise training regimens in old individuals [80]. Moreover, in older individuals, it has been reported that treatment with COX inhibitors improves the exercise-induced enhancement of muscle mass and strength (25%–50%) over a placebo-consuming group [11,12,81]. These responses seem to be mediated through the reduction of the PGE_2_-IL-6-MuRF-1 levels in the muscle with the COX inhibitors [83], which result in an inhibition in protein degradation. This is accompanied with an up-regulation of the PGF_2α_ receptor in the muscle of the treated groups [12]. This increase coupled with a training increase in COX-1, PGF_2α_ synthase and PGE_2_-to-PGF_2α_ reductase, would make the muscle more sensitive to PGF_2α_ produced following exercise [11].

The animal models of hypertrophy have been criticized because their characteristics differ significantly from the human models [11]. This is why we have focused our review in the human studies. However, we would like to mention a recent study in rats in which the authors found that chronic consumption of ibuprofen limited sarcopenia through restoration of the muscle protein-synthesis response in the post-prandial state in older rats [84]. Mechanisms leading to sarcopenia involve the imbalance between rates of protein synthesis and degradation that is detected in the postprandial state in old animals and humans [84]. During aging, low-grade inflammation (a slight increased cytokine production) exerts a deleterious effect on muscle protein synthesis stimulation and muscle proteolysis inhibition by food intake. Thus, by decreasing the levels of IL-6 and IL-1β with chronic treatment with ibuprofen it has been shown that muscle mass loss can be prevented in old animals [84]. These results open up the possibility of treating sarcopenia with COX-inhibiting drugs.

## 4. Conclusions

As a summary we would like to highlight three main points: (1) COX-inhibiting drugs modulate the protein synthesis response to resistance exercise and to training differently; (2) contrary to what has been reported with antioxidants, chronic consumption of COX inhibitors during exercise training does not appear to interfere with muscle mass and strength gains expected from resistance-exercise-training regimens; and (3) recent studies open up the possibility of treating sarcopenia with COX-inhibiting drugs.

## Figures and Tables

**Table 1:** Summary of the human studies of PGs, skeletal muscle, and exercise responses and/or adaptations.

Author	Sample	Training Protocol	Dose of the Anti-Inflammatory	Main Findings
Trappe and co-workers [9]	Humans (Young)	10–14 sets of 10 eccentric repetitions at 120% of concentric one repetition maximum with the knee extensors	Ibuprofen (1200 mg/day), acetaminophen (4000 mg/day) after the exercise	Anti-inflammatory attenuated the increased rate of muscle protein synthesis 24 h after exercise. No effect on whole body protein breakdown, on serum CK, or on muscle soreness
Krentz and co-workers [79]	Humans (Young)	6 sets of biceps curls (3 sets of 8 to 10 concentric repetitions at 70% 1 RM and 3 sets of 4 to 6 eccentric repetitions at 100% 1 RM), 2–3 days/week for 6 week	Ibuprofen (400 mg) taken after exercise	No effect of ibuprofen on muscle growth or strength adaptations
Petersen and co-workers [80]	Human (Old with osteoarthritis)	12 week of progressive resistance training, 3 days/week (4 to 5 sets of 8 to 15 repetitions at 70%–80% of 1 RM)	Ibuprofen (1200 mg/day)	No effect on muscle mass gains, but muscle strength was enhanced in those individuals consuming the COX inhibitor (maybe explained by the pain relief).
Trappe and co-workers [11,12,81]	Human (Old healthy)	Resistance-exercise training 3 days/week (3 sets of 10 repetitions at 75% of 1 RM/day) for 12 week	Acetaminophen (4000 mg/day) or ibuprofen (1200 mg/day)	COX inhibitors enhance muscle mass and strength gains of 25%–50% over placebo: Reduced the PGE_2_-IL-6-MuRF-1 levels in skeletal muscle. Upregulated PGF_2α_ receptor and its protein synthesis effect
Trappe and co-workers [82]	Human (Old healthy)	12 weeks of knee-extensor resistance exercise (3 days/week)	Acetaminophen (4000 mg/day)	COX inhibitor enhances myocellular growth, and this effect is more pronounced in Type I muscle fibers
